# A developmental, longitudinal investigation of autism phenotypic profiles in fragile X syndrome

**DOI:** 10.1186/s11689-016-9179-0

**Published:** 2016-12-30

**Authors:** Michelle Lee, Gary E. Martin, Elizabeth Berry-Kravis, Molly Losh

**Affiliations:** 1 Department of Psychiatry and Behavioral Sciences, Northwestern University Feinberg School of Medicine, Chicago, IL USA; 2 Department of Communication Sciences and Disorders, St. John’s University, Staten Island, NY USA; 3 Rush University Medical Center, Chicago, IL USA; 4 Department of Communication Sciences and Disorders, Northwestern University, Evanston, IL USA

**Keywords:** Autism spectrum disorder, Fragile X syndrome, Endophenotype, Longitudinal, Social behavior, Language, Pragmatics, *FMR1* gene

## Abstract

**Background:**

Targeting overlapping behavioral phenotypes in neurogenetic disorders can help elucidate gene-behavior relationships. Fragile X syndrome (FXS) and autism spectrum disorder (ASD) have been studied as a model for this approach, and important areas of phenotypic overlap and divergence have been documented. However, few studies have examined how the manifestation of ASD-related phenotypes in FXS may change over development, a question which has important implications for conceptualizing shared etiologies of these disorders and their constituent phenotypes. The goal of this study was to characterize ASD phenotypes in boys and girls with FXS across development, as well as to compare individual component phenotypes among boys with FXS and boys with idiopathic ASD (ASD-O) over time.

**Methods:**

Sixty-five boys and girls with FXS and 19 boys with ASD-O completed a battery of diagnostic, cognitive, and language assessments at two time points (mean 2.5 years apart). Nonparametric tests assessed changes in diagnostic classification in FXS over time, and hierarchical linear modeling and repeated measures assessed changes in individual ASD symptoms in FXS over time. Additionally, ANCOVAs compared ASD symptom severity and component phenotypes in boys with FXS-O, FXS-ASD, and ASD-O at both time points.

**Results:**

Overall, ASD symptom manifestation for children with FXS significantly increased over time, and developmental predictors varied based on the domain of symptoms assessed. The greatest degree of overlap was observed between boys with FXS-ASD and ASD-O in the domain of reciprocal social communication across time points, whereas boys with ASD-O demonstrated greater impairment in restricted and repetitive behaviors at the later time point.

**Conclusions:**

ASD symptoms increased in FXS with age, and social language impairment emerged as a potential core shared feature of FXS and ASD that may help elucidate underlying molecular genetic variation related to phenotypic variance, and aid intervention planning for subgroups of children showing distinct phenotypes. Results highlight the value of a developmental perspective, and longitudinal data in particular, in evaluating shared behavioral phenotypes across genetic conditions, lending insight into underlying cognitive, neural, and genetic mechanisms associated with key developmental phenotypes in ASD and FXS.

## Background

Since the development of psychiatric nosology, it has been recognized that clinical symptoms are often shared across categorically defined disorders. Consistent with clinical observations, multiple genome-wide analysis studies (GWAS) have produced molecular evidence of shared genetic risk factors in several psychiatric disorders [35, 50]. Clearly defining phenotypes cutting across diagnostic boundaries may help to clarify relationships between behavioral symptoms and underlying biology with long-term implications for individualized treatment; this approach has been proposed as a primary direction for the future of psychiatric research [29]. A critical consideration in such efforts is how psychiatric phenotypes may manifest differently across development. Longitudinal research on disorders such as attention deficit-hyperactivity disorder [52], Down syndrome [44], and autism spectrum disorder (ASD; [14, 62]) demonstrate changes in clinical symptoms and underlying neurobiology across the lifespan, underscoring the importance of examining development when characterizing symptomatology within and across different psychiatric disorders. Yet, few studies have adopted a developmental perspective when examining shared genetic liability across conditions. This paper examines developmental changes in diagnostic classification, symptom expression, and related abilities in fragile X syndrome (FXS) and ASD, two complex neurodevelopmental disorders with considerable phenotypic overlap. At both behavioral and neurobiological levels, FXS and ASD are characterized by atypical developmental trajectories. Therefore, charting the dual paths of phenotypic development in each disorder can help to identify markers of shared etiology throughout the lifespan, with direct clinical, methodological, and theoretical implications.

ASD is characterized by social and communicative impairments and restricted and repetitive behaviors or interests [2]. Hundreds of copy-number variations (CNVs), de-novo mutations, and individual loci conferring elevated risk have been implicated in ASD [56]. Monogenic disorders account for up to 20% of diagnosed cases of ASD, the most common of which is FXS [63]. FXS is caused by a Cytosine-Guanine-Guanine (CGG) repeat expansion of over 200 in the promoter region of the fragile X mental retardation 1 (*FMR1*) gene on the X chromosome. This expansion leads to methylation of the promoter and effective silencing of the *FMR1* gene, resulting in significant reduction or loss of fragile X mental retardation protein (FMRP), an essential protein for brain development and, in particular, the regulation of synaptic function (see [4], for review). Individuals with FXS present with heterogeneous cognitive, language, social, and behavioral deficits [1, 4, 6]. Additionally, over 90% of individuals with FXS display some ASD symptoms [28].

This considerable phenotypic overlap, together with recognition that ASD appears to arise from heterogeneous molecular genetic causes, has prompted investigations of FXS (along with other monogenic conditions overlapping with ASD) as a paradigm for understanding gene-brain-behavior relationships relevant to ASD symptomatology in a simplified genetic context [7, 22, 54]. FMRP is an inhibitor of dendritic translation, involved in the regulation of synaptic development, plasticity, and activity, making it a good candidate for involvement in symptoms associated with ASD given evidence implicating synaptic disruptions in ASD [45, 55]. Specifically, FMRP suppresses group 1 metabotropic (mGluR1, mGluR5) glutamate receptor-regulated translation, thereby regulating numerous genes implicated in ASD (i.e., *NLGN3, NRXN, SHANK3, PTEN, TCS2,* and *NF*1; [5, 15, 59]). Of note, inhibition of mGluR5 in the FXS mouse model has been shown to normalize numerous abnormal synaptic, signaling, electrophysiological, and behavioral phenotypes that overlap with ASD [45]. Finally, reduced levels of FMRP in FXS have also been shown to disrupt signaling pathways of neurotransmitters implicated in ASD, such as dopaminergic receptors [43], GABA [12], and 5-HT [30].

Detailed characterization of shared phenotypes in these disorders is necessary to identify specific behaviors that may map more directly onto common underlying biology (i.e., endophenotypes). Family studies of ASD indicate that endophenotypes segregate independently in unaffected relatives, possibly reflecting unique genetic contributions [38, 47]. Therefore, characterizing ASD phenotypes in the context of a monogenic disorder such as FXS offers a unique opportunity to identify endophenotypes related to known genetic variation. Social deficits represent the most consistent area of overlap between idiopathic ASD and individuals with FXS with comorbid ASD (i.e., FXS-ASD) in both severity and quality [8, 31–33, 53]. For instance, Klusek et al. [33] found that boys with idiopathic ASD and FXS-ASD demonstrated highly similar pragmatic (i.e., social) language profiles that were not accounted for by overall cognitive impairment. Further, it has been reported that *FMR1* premutation carriers (i.e., CGG repeat length between 55 and 200) may evidence higher rates of ASD [3, 18, 19, 57], though population-based studies are still needed, and clinically unaffected carriers also demonstrate subtle pragmatic difficulties similar to those observed among unaffected relatives of individuals with ASD [40]. Therefore, social language may be a promising candidate ASD endophenotype connected with *FMR1*-related variation.

Not all ASD phenotypes express similarly among individuals with FXS, and understanding such differences is equally informative in determining common pathways between the two disorders. For instance, within the domain of restricted and repetitive behaviors (RRBs), careful examination of the *types* of behaviors demonstrated across groups (rather than overall frequency) indicated similar rates of lower level motoric RRBs in FXS and idiopathic ASD but fewer higher order compulsive and repetitive behaviors in individuals with FXS meeting criteria for ASD [61]. A mix of similarities and differences has also been reported in the biophysiological and neuroanatomical profiles of ASD and FXS [27, 41, 46, 62]. Mapping areas of convergence and divergence in FXS and ASD is therefore essential for deconstructing the heterogeneity of ASD, identifying those phenotypes that may relate to *FMR1*, and guiding clinical interventions for both disorders.

Adopting a developmental perspective in such efforts is critical for accounting for phenotypic changes that occur with children’s growth and maturation. Although pronounced changes are known to occur in such core ASD symptom domains as social communication over time, few studies have examined the role of development in studies of ASD symptom manifestation in FXS, despite the fact that studies conducted at a single time point have included considerable age ranges (5–60 years; [34]). Existing findings from cross-sectional work suggests variation in the expression of phenotypes associated with ASD in children with FXS at different ages and cognitive abilities [25, 34, 42, 53, 58], whereas longitudinal approaches using questionnaires or behavioral ratings suggest more stability of ASD symptoms in FXS [13, 26, 51]. In the one longitudinal study using a gold-standard clinical measure of ASD symptoms, Hernandez et al. [28] found that approximately one third of their FXS sample demonstrated inconsistent ASD classification over time based on the administration of the Autism Diagnostic Interview-Revised (ADI-R; [37]), a comprehensive parent-interview, although this change was not statistically significant. Therefore, further direct-assessment evaluations of ASD phenotypes in FXS are needed to evaluate key phenotypes that overlap and to identify specific developmental factors that impact the manifestation of ASD symptoms in FXS, in order to advance clinical, methodological, and theoretical approaches.

This study examined the developmental manifestation of ASD phenotypes in FXS, drawing from comprehensive longitudinal assessments of children with FXS and idiopathic ASD (i.e., ASD-O) using gold-standard ASD diagnostic measures, and standardized assessments of cognition, structural language (i.e., vocabulary), and pragmatic language. We aimed to (1) characterize trajectories of ASD symptoms in FXS and predictors of ASD symptoms over time and (2) compare the type and severity of symptoms and individual behaviors observed in boys with FXS to a group of boys with ASD-O.

## Methods

### Participants

Participants included 65 children with FXS (31 male, 34 female) and 19 boys with ASD-O. Because FXS is a rare disorder, 12 pairs of siblings were included in analyses to maximize sample size (see Table 1 for participant characteristics). All participants spoke English as their first language and were screened for use of at least three-word phrases. Inclusion criteria for boys with ASD-O included a previous clinical diagnosis confirmed through direct assessment with the Autism Diagnostic Observation Schedule (ADOS; [36]) and/or the ADI-R [37], and no known ASD-related monogenic disorders.

**Table 1:** Overall sample characteristics at time 1 and time 2

Time one	Group	*n*	Chronological age M (SD)	Nonverbal mental age M (SD)	EVT age equivalent M (SD)	PPVT age equivalent M (SD)	ADOS module distribution
	*FXS-girls*	*34*	*8.96 (3.39)*	*6.18 (1.72)* ^*a*^	*6.95 (2.67)* ^*a*^	*7.40 (3.06)* ^*a*^	*14 M2, 20 M3*
	FXS-O	24	8.50 (3.37)	6.51 (1.87)	7.32 (2.75)	7.90 (3.12)	11 M2, 13 M3
	FXS-ASD	10	9.11 (3.14)	5.39 (.99)	6.05 (2.40)	6.98 (3.81)	3 M2, 7 M3
	*FXS-boys*	*31*	*8.97 (2.51)*	*4.77 (.69)* ^*b*^	*4.69 (1.23)* ^*b*^	*5.46 (1.53)* ^*b*^	*2 M1, 18 M2, 11 M3*
	FXS-O	14	8.47 (2.58)	4.79 (.75)	4.51 (2.57)	5.19 (1.43)	1 M1, 8 M2, 5 M3
	FXS-ASD	17	9.38 (2.45)	4.75 (.66)	4.84 (1.21)	5.67 (1.62)	1 M1, 10 M2, 6 M3
	*ASD (boys)*	*19*	*9.08 (2.31)*	*5.82 (1.43)* ^*b*^	*5.79 (1.62)* ^*b*^	*5.85 (1.71)* ^*b*^	*2 M2, 11 M3*
Time two	Group	*n*	Chronological age M (SD)	Nonverbal mental age M (SD)	EVT age equivalent M (SD)	PPVT age equivalent M (SD)	ADOS module distribution
	*FXS-girls*	*34*	*11.21 (3.31)*	*7.31 (3.04)* ^*a*^	*8.50 (2.95)* ^*a*^	*9.48 (3.55)* ^*a*^	*2 M2, 31 M3, 1 M4*
	FXS-O	20	11.98 (3.44)	8.12 (3.60)	9.61 (2.84)	10.88 (3.49)	20 M3
	FXS-ASD	13	10.12 (2.88)	6.06 (1.18)	6.88 (2.35)	7.33 (2.46)	2 M2, 11 M3, 1 M4
	*FXS-boys*	*31*	*11.50*	*5.01(.50)* ^*b*^	*5.36(1.27)* ^*b*^	*6.15(1.51)* ^*b*^	*5 M2, 26 M3*
	FXS-O	6	10.78 (1.65)	5.17 (.73)	6.06 (1.50)	6.71(.64)	6 M3
	FXS-ASD	25	11.67 (2.5)	4.98 (.44)	5.19 (1.18)	6.02 (1.64)	5 M2, 20 M3
	*ASD (boys)*	*19*	*11.38 (2.63)*	*6.93(1.84)* ^*b*^	*6.79 (2.14)* ^*b*^	*7.68(2.11)* ^*b*^	*2 M2, 17 M3*

Differing superscripts indicate groups which significantly differed overall (*p* < .05). “M” represents ADOS module type. Italicized data indicate overall means by sex. FXS-O and FXS-ASD group classification changed over time based on behaviors observed during the ADOS. Five participants with FXS had missing covariate data

### Procedures

At two time points (mean 2.5 years apart, range 1.15–3.90 years) participants completed a battery of cognitive, language, and ASD diagnostic measures. Because this sample was selected to assess stability over time, only participants with greater than one time point were included in the analyses. Participants were recruited through advertisements at genetic clinics and physicians’ offices, advocacy groups, and participant registries. All participants provided informed consent and the University of North Carolina at Chapel Hill (IRB #07-0044) and Northwestern University Institutional Review Boards (IRB # STU00039816) approved these procedures.

### Measures

#### ASD classification

Participants were administered the ADOS and the ADI-R. The ADOS is a standardized, semi-structured assessment that includes a range of activities designed to elicit social interaction and consists of four different modules that account for differences in developmental level and language abilities. The ADOS determined ASD classification for children with FXS and confirmed ASD classification for boys with ASD-O. Classification was based on revised algorithms of the ADOS, and continuous symptom severity scores were also determined [20, 21]. Consistent with DSM-5 [2], participants with FXS that met either “spectrum” or “autism” criteria on the ADOS were classified as FXS-ASD. The ADOS consists of a range of individual items rated on a scale of 0–3 (absent–severe). In order to compare performance on items across modules, codes that were identical or tapping the same symptoms across modules were identified and used in item-level analyses. For these analyses, items rated an 8 or 9 (i.e., a code was not applicable or there was an administration error) were reduced to 0, consistent with algorithm coding. The ADI-R is a parent interview that assesses early developmental features and provides an algorithm to determine ASD classification. The ADI-R was administered to confirm ASD in participants with ASD-O and to examine classification agreement with the ADOS in individuals with FXS over time.

#### Cognitive and language abilities

The Leiter International Performance Scale-Revised [49] provided an estimate of nonverbal mental age. The Expressive Vocabulary Test (EVT; [60]) and Peabody Picture Vocabulary Test-3rd or 4th edition (PPVT; [16, 17]) were used to assess expressive and receptive language. To assess pragmatic language, participants completed the Pragmatic Judgment subtest of the Comprehensive Assessment of Spoken Language (CASL; [9]), which evaluates awareness of appropriate language use in various social situations (e.g., how to greet an unfamiliar adult or how to give an appropriate compliment).

### Analysis plan

The first aim of this study was to characterize trajectories of ASD symptoms in FXS and predictors of ASD symptoms over time. McNemar’s test of classification assessed whether rates of ASD and agreement between ADOS and ADI-R changed over time. Nonparametric Wilcoxon signed-rank tests were used to assess changes in individual ADOS items across time points. These analyses were run separately by sex and also within the group of boys and girls with FXS who demonstrated a change in ASD classification on the ADOS. Finally, hierarchical linear models, nesting age within participant, were completed to assess the statistical effect of chronological age, sex, mental age, receptive and expressive vocabulary, and pragmatic judgment age equivalence on measures of symptom severity derived from the ADOS across time points. To reduce collinearity among predictors, all predictor variables in these models were mean-centered and expressive and receptive vocabulary measures were combined into a composite by summing the raw score on each measure.

The second aim of this study was to compare the severity and type of ASD symptoms in boys with FXS to a group of boys with ASD-O at each time point. ANCOVAs, controlling for mental age and receptive and expressive vocabulary age equivalence, followed by planned comparisons, compared ASD severity scores as well as individual items at both time points for boys classified as FXS-O, FXS-ASD, and ASD-O. Although item-level scores are ordinal in nature, this parametric statistical approach was chosen in order to include mental age and vocabulary as covariates. Thus, results of item-level comparisons should be interpreted cautiously.

## Results

### Aim one: characterize trajectories of ASD symptoms in FXS and predictors of ASD symptoms over time

Overall, 41.7% of children with FXS met criteria for ASD at time one (54.8% of boys, 41.5% of girls); at time two, 60% of the sample met criteria (80.6% of boys, 41.2% of girls), a statistically significant change (*p* = .008), driven by the change in classification in boys with FXS (*p* = .021). There was a nearly significant increase in ADI-R and ADOS agreement over time (*p* = .065), driven by a significant increase in agreement for boys with FXS (45.2 to 75%, *p* = .039), whereas agreement for girls with FXS decreased slightly (73.5 to 64.7%, *p* = .45).

Repeated measure analyses were then conducted for individual items on the ADOS. Boys with FXS overall demonstrated increased impairments in the area of shared enjoyment (*Z* = −2.57, *p* = .010) and social overtures (*Z* = −2.71, *p* = .007), and girls with FXS demonstrated an increase in impairments in prosodic features of speech (*Z* = −2.42, *p* = .016), facial expressions (*Z* = −2.04, *p* = .041), and social overtures (*Z* = −2.71, *p* = .05). No other significant changes were noted on any other item in these groups (*Z*s > −1.90, *p*s > .05). Item level analyses were replicated within the group of participants who did not meet ASD criteria at time one but did at time two in order to assess symptoms that worsened with time. Within this group, several algorithm items (i.e., items that would contribute to a classification change) increased in impairment: conversation (*Z* = −2.1, *p* = .035), facial expressions (*Z* = −2.31, *p* = .021), social overtures (*Z* = −2.95, *p* = .003), social response (*Z* = −2.83, *p* = .005), and quality of rapport (*Z* = −2.11, *p* = .035). In addition, impairment on a non-algorithm item, atypical qualities of speech (*Z* = −1.98, *p* = .048) also increased. No other significant changes were noted (*Z*s > −1.83, *p*s > .068).

Hierarchical linear models revealed a significant main effect of age, in that greater age was associated with greater symptom severity for all measures of severity (see Table 2). Additionally, pragmatic competence was marginally associated with reduced overall severity and severity of social affect (i.e., for every year increase in pragmatic language age equivalence, predicted overall symptom severity decreased by .34 and severity of social affect decreased by .32). In contrast, for restricted and repetitive behaviors, mental age significantly predicted decreases in severity (i.e., for every year increase in age equivalence, severity decreased by .38).

**Table 2:** Main effects of hierarchical linear models

	Test
Overall severity	
Chronological age	*F*(1,88.41) = 17.31, *p* < .001
Sex	*F*(1,64.60) = .00, *p* = .98
Chronological age*sex	*F*(1,104.92) = .404, *p* = .53
Mental age	*F*(1,102.26) = 1.59, *p* = .211
Receptive and expressive vocabulary	*F*(1,109.50) = .142, *p* = .71
Pragmatic language	*F*(1,114.85) = 3.81, *p* = .054
Severity of social affect	
Chronological age	*F*(1,85.7) = 17.03, *p* < .001
Sex	*F*(1,64.21) = .07, *p* = .80
Chronological age*sex	*F*(1,101.32) = .43, *p* = .51
Mental age	*F*(1,98.02) = .49, *p* = .49
Receptive and expressive vocabulary	*F*(1,106.74) = .35, *p* = .56
Pragmatic language	*F*(1,115.88) = 3.45, *p* = .07
Severity of restricted and repetitive behavior	
Chronological age	*F*(1,83.67) = 6.36, *p* = .014
Sex	*F*(1,66.56) = .019, *p* = .87
Chronological age*sex	*F*(1,96.37) = 1.65, *p* = .20
Mental age	*F*(1,92.45) = 4.78, *p* = .03
Receptive and expressive vocabulary	*F*(1,102.35) = .02, *p* = .89
Pragmatic language	*F*(1,115.17) = 3.06, *p* = .08

* represents the interaction between chronological age and sex

### Aim two: comparisons of symptom severity and type of ASD symptoms in boys with FXS and ASD-O over time

**Fig. 1:**
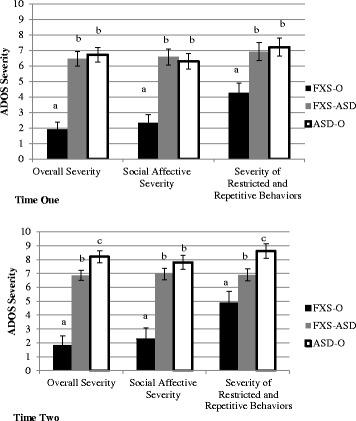
Comparison of ASD symptom severity of boys with FXS-O, FXS-ASD, and ASD-O at each time point. Notes: ADOS severity derived from Gotham et al. [21]. Differing letters convey significant differences at the level of *p* < .05. Means are adjusted for nonverbal mental age, expressive vocabulary, and receptive vocabulary

At time one, overall ASD symptom severity and severity of social impairment and RRBs in boys with FXS-ASD were nearly indistinguishable from boys with ASD-O (*p*s > .50; see Figure 1), and significantly greater than boys with FXS-O (*F*(2,43) = 33.57, *p* < .001; *F*(2,43) = 23.81, *p* < .001; *F*(2,43) = 7.36, *p* = .002). At time two, a stepwise pattern was observed in which boys with ASD-O demonstrated significantly greater overall severity (driven by more severe RRBs) than boys with FXS-ASD, who in turn displayed greater severity than boys with FXS-O (*F*(2,44) = 30.55, *p* < .001; *F*(2,44) = 6.86, *p* = .003). Boys with ASD-O did not differ from boys with FXS-ASD on severity of social impairment at time two (*p* = .26), and both were significantly greater than boys with FXS-O (*F*(2,44) = 17.98, *p* < .001).

**Fig. 2:**
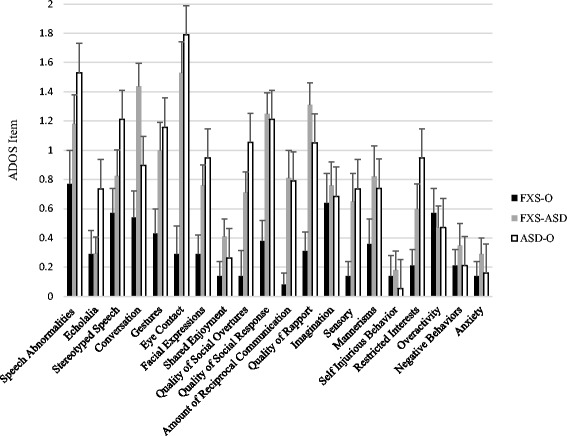
Profile of ASD phenotypic expression on ADOS items at time one. Notes: Significant differences are reported in text. After accounting for mental age, and receptive and expressive vocabulary, males with FXS-ASD and ASD-O demonstrated overlap on several items tapping social communication, whereas individuals with ASD-O demonstrated greater impairment in restricted interests, gestures and echolalia

**Fig. 3:**
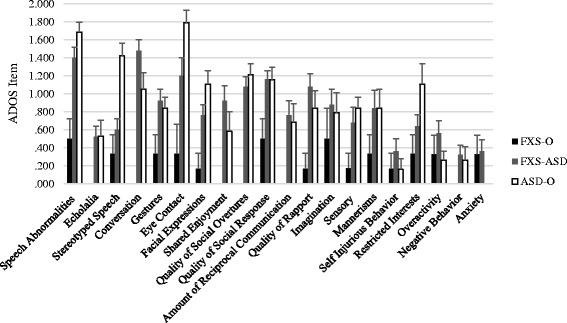
Profile of ASD phenotypic expression on ADOS items at time two. Notes: Significant differences are reported in text. After accounting for mental age, and receptive and expressive vocabulary, males with FXS-ASD and ASD-O demonstrated overlap on several items tapping social communication, whereas individuals with ASD-O demonstrated greater impairment in restricted interests

Figures 2 and 3 demonstrate profiles of performance on individual ADOS items across boys with FXS-O, FXS-ASD, and ASD-O. At time one, groups did not differ in items tapping prosodic features of speech, shared enjoyment, imagination, sensory seeking behaviors, repetitive motor movements, self-injury, overactivity, negative behavior, or anxiety (*F*(2,41) < 2, *p*s > .05). However, several significant group differences emerged (overall model *F*(2,41) > 3.7, *p*s < .05). Boys with ASD-O and FXS-ASD demonstrated overlap in conversation, eye contact, quality of social overtures and responses, amount of reciprocal communication, and overall quality of rapport (*p*s > .05), and significantly greater impairment than boys with FXS-O across these domains (*p*s < .05). Boys with ASD-O demonstrated greater impairments in restricted interests, gestures, and echolalia relative to both FXS-ASD and FXS-O groups (*p*s < .05), who did not differ from one another (*p*s > .07), and boys with ASD also demonstrated greater impairments in facial expressions and stereotyped speech than boys with FXS-O only (*p*s < .03).

At the second time point, groups did not differ on items tapping echolalia, gestures, shared enjoyment, imagination, sensory seeking behaviors, repetitive motor movements, self-injurious behavior, overactivity, negativity, or anxiety (*F*(2,44) < 2.9, *p*s > .06); group differences emerged on the remaining items (overall *F*(2,44) > 3.3, *p*s < .05). Boys with FXS-ASD and ASD-O did not differ from each other (*p*s > .06), and both demonstrated greater impairment than boys with FXS-O in the domains of prosodic features of speech, conversation, eye contact, quality of social response, amount of reciprocal social interactions, and overall quality of rapport (*p*s < .05). A stepwise pattern was observed for facial expressions and social overtures in which boys with FXS-O demonstrated less impairment than boys with FXS-ASD, who in turn demonstrated less impairment than boys with ASD-O (*p*s < .05). Finally, boys with ASD-O demonstrated greater impairments in stereotyped speech and restricted interests than boys with FXS-ASD and FXS-O (*p*s < .01).

## Discussion

This study applied a longitudinal lens to characterize ASD symptom profiles in the context of FXS and identify potential overlapping endophenotypes in ASD and FXS across development. Findings suggest ASD symptoms worsen with age and that this increase is most prominently observed in social communication skills. Substantial phenotypic overlap, as well as key areas of difference, was observed in boys with ASD-O and FXS-ASD at both time points.

In contrast to prior longitudinal reports (e.g., [26, 28]), ASD symptom manifestation in FXS was variable over time, with both classification and symptom severity significantly increasing over time. This study was the first to use the ADOS to examine changes over time; it may be that direct assessments of behavior better capture phenotypic variation contributing to diagnostic status than parent report, in line with Harris et al. [24] finding that the ADOS classifications were most consistent with DSM-IV diagnoses. Of note, Klusek et al. [34] examined ADOS and ADI-R agreement in a cross-sectional sample overlapping with the current study and thus similar agreement rates are not surprising. Rather, findings from the current study build on this prior work by suggesting that agreement increases with age, particularly for males with FXS. Consistent with prior work showing that ASD is more common in males than females with FXS [11, 23, 34], girls with FXS in the current sample continued to demonstrate reduced rates of ASD classification relative to boys over time, although sex did not impact rates of change over time when controlling for mental age and structural language. Whereas studies should continue to investigate the role of assessment approach and sex in observed changes in symptoms over time, findings demonstrating an increase in ASD symptoms with age have important implications for long-term intervention planning for children with FXS, as well as future research examining overlapping profiles of individuals with ASD and FXS.

The longitudinal approach of this study also allowed for analyses of developmental features related to ASD symptoms in individuals with FXS over time. The roles of nonverbal mental age, structural language (i.e., receptive and expressive vocabulary) abilities, and pragmatic competence in ASD symptom presentation in FXS changed as a function of the domain assessed. For example, when examining the significance of mental age in predicting symptom severity, significant relationships emerged for severity of restricted and repetitive behaviors, consistent with prior findings from cross-sectional work [42, 58] and group comparisons at a single time point (e.g., [23]). However, mental age did not predict severity of social-affective symptoms. Thus, while general cognition remains an important consideration when examining the expressions of ASD phenotypes in FXS, lower mental age does not appear to account for ASD symptoms in FXS entirely. Instead, variation in pragmatic competence was the only significant covariate in the model examining predictors of severity of social affect over time, further suggesting the importance of pragmatic development in observed impairments in FXS.

The language demands of the assessment context may also play a role in observed ASD symptoms in FXS. In fact, whereas at baseline approximately 50% of individuals with FXS received a module 2 of the ADOS (a version that is less linguistically demanding), with development of language over 80% of individuals with FXS received a module 3 at the later time point. That is, assessments administered at time one involved limited expressive language requirements whereas assessments at time two entailed a heavier emphasis on expressive language ability and reciprocal conversation (i.e., ADOS module change). Therefore, the development of language abilities may afford more observable manifestations of ASD-related social and communicative deficits in children with FXS.

Interestingly, in the subgroup of children with FXS who demonstrated a change in classification over time, the phenotypes driving this change were primarily related to social interaction, including conversation, amount of reciprocation, and social responses. Indeed, across time points, social impairments emerged as a core shared phenotype across boys with FXS-ASD and ASD-O, with highly similar levels of social affective severity as well as several items linked to social interactions (e.g., social overtures and responses) observed in both children with FXS-ASD and ASD-O across time points. These findings add to a growing body of work demonstrating that communication [31] and reciprocal social behaviors are shared across FXS-ASD and ASD-O [8, 31, 32], predict later ASD classification [48], and align in particular with literature showing a high degree of pragmatic impairment overlap in FXS-ASD and ASD-O [33, 40] as well as similarities in pragmatic language profiles documented among relatives who are genetic carriers [39]. An important next step will be to continue to investigate mechanisms underlying this overlap across groups; for example, Roberts et al. [48] found that differential HPA axis activation (i.e., a measure of the body’s response to stress) impacted social approach behaviors in individuals with FXS with and without comorbid ASD. Such investigations, targeting specific aspects of social behaviors across development, hold promise for continuing to elucidate ASD endophenotypes related to *FMR1* variation.

Some important differences between boys with ASD-O and FXS-ASD also emerged across time points. For example, the FXS-ASD group did not differ in severity of RRBs at time one but did differ at the later time point. Important to note is that this difference was driven by the fact that boys with ASD-O demonstrated significantly greater impairments in restricted interests (a higher order RRB) in particular. This difference may therefore have been influenced by a distinct profile of RRBs in FXS-ASD relative to ASD-O, as reported in prior work [61, 62], and that may be more clearly observed with age. Further, males with ASD-O demonstrated significantly greater impairments than those with FXS-ASD on symptom domains tapped by the ADOS that varied across time points, in that at time one, but not at time two, boys with ASD-O demonstrated greater rates of gestures and echolalia. Such findings affirm that ASD in FXS is not identical to that observed in ASD-O, that age is likely to influence manifestation of this overlap, and that specific target behaviors, such as social responses, may best map onto underlying shared genetic etiology.

Together, results suggest that ASD as it presents in FXS is not identical to idiopathic ASD. Given the clinical and etiologic complexities of each disorder, this is not necessarily surprising, nor should it undermine the utility of studying FXS and other monogenic conditions as models for understanding ASD related phenotypes. In line with recent conceptualizations of psychiatric nosology, and complementary findings from psychiatric genomics, current findings strongly suggest that mapping homogeneous sets of phenotypes cutting across disorders (and ultimately, related underlying neural and molecular mechanisms, and their interplay with environmental influences) is crucial to understanding the pathogenesis of complex psychiatric disorders such as ASD [10, 29]. Findings here suggest that adopting a developmental framework in such comparisons can provide necessary sharpened focus to the analysis of complex phenotypes across disorders. In the case of FXS and ASD, it appears that social-communicative behaviors may be particularly fruitful targets for biological study and clinical intervention, where shared phenotypes can serve as a basis for translating new findings and treatments across disorders to inform clinical and research practice.

## Conclusions

Current findings highlight the importance of adopting a developmental perspective when investigating shared behavioral features across disorders. Including assessments across multiple time points allowed for the identification of overlapping and divergent phenotypes across two complex neurodevelopmental disorders and clarification for the role of related abilities in observed symptoms over time, with key implications for understanding etiologic mechanisms related to shared phenotypes across ASD and FXS, and implications for clinical intervention. Incorporating a developmental perspective within a shared phenotype approach will continue to inform the search for genetic etiology of psychiatric disorders, with potential for shaping clinical and research practice.
