# Erratum to: A developmental, longitudinal investigation of autism phenotypic profiles in fragile X syndrome

**DOI:** 10.1186/s11689-017-9192-y

**Published:** 2017-03-06

**Authors:** Michelle Lee, Gary E. Martin, Elizabeth Berry-Kravis, Molly Losh

**Affiliations:** 1 Department of Psychiatry and Behavioral Sciences, Northwestern University Feinberg School of Medicine, Chicago, IL USA; 2 Department of Communication Sciences and Disorders, St. John’s University, Staten Island, NY USA; 3 Rush University Medical Center, Chicago, IL USA; 4 Department of Communication Sciences and Disorders, Northwestern University, Evanston, IL USA

## Erratum

During production of article [1], Table 1 was generated with incorrect data. The changes applied are listed below:

**Table 1:** Overall sample characteristics at time 1 and time 2

**Time One**	Group	n	Chronological Age M (SD)	Nonverbal Mental Age M (SD)	EVT Age Equivalent M (SD)	PPVT Age Equivalence M (SD)	ADOS Module Distribution
	**FXS-Girls**	**34**	**8.96 (3.39)**	**6.18 (1.72)** ^**a**^	**6.95 (2.67)** ^**a**^	**7.40 (3.06)** ^**a**^	**14 M2, 20 M3**
	FXS-Only	24	8.50 (3.37)	6.51 (1.87)	7.32 (2.75)	7.90 (3.12)	11 M2, 13 M3
	FXS-ASD	10	9.11 (3.14)	5.39 (.99)	6.05 (2.40)	6.98 (3.81)	3 M2, 7 M3
	**FXS-Boys**	**31**	**8.97 (2.51)**	**4.77 (.69)** ^**b**^	**4.69 (1.23)** ^**b**^	**5.46 (1.53)** ^**b**^	**2 M1, 18 M2, 11 M3**
	FXS-O	14	8.47 (2.58)	4.79 (.75)	4.51 (2.57)	5.19 (1.43)	1 M1, 8 M2, 5 M3
	FXS-ASD	17	9.38 (2.45)	4.75 (.66)	4.84 (1.21)	5.67 (1.62)	1 M1, 10 M2, 6 M3
	**ASD (Boys)**	**19**	**9.08 (2.31)**	**5.82 (1.43)** ^**b**^	**5.79 (1.62)** ^**b**^	**5.85 (1.71)** ^**b**^	**8 M2, 11 M3**
**Time Two**	Group	n	Chronological Age M (SD)	Nonverbal Mental Age M (SD)	EVT Age Equivalent M (SD)	PPVT Age Equivalence M (SD)	ADOS Module Distribution
	**FXS-Girls**	**34**	**11.21 (3.31)**	**7.31 (3.04)** ^**a**^	**8.50 (2.95)** ^**a**^	**9.48 (3.55)** ^**a**^	**2 M2, 31 M3, 1 M4**
	FXS-Only	20	11.98 (3.44)	8.12 (3.60)	9.61 (2.84)	10.88 (3.49)	20 M3
	FXS-ASD	**14**	10.12 (2.88)	6.06 (1.18)	6.88 (2.35)	7.33 (2.46)	2 M2, 11 M3, 1 M4
	**FXS-Boys**	**31**	**11.50 (2.37)**	**5.01(.50)** ^**b**^	**5.36(1.27)** ^**b**^	**6.15(1.51)** ^**b**^	**5 M2, 26 M3**
	FXS-O	6	10.78 (1.65)	5.17 (.73)	6.06 (1.50)	6.71(.64)	6 M3
	FXS-ASD	25	11.67 (2.5)	4.98 (.44)	5.19 (1.18)	6.02 (1.64)	5 M2, 20 M3
	**ASD (Boys)**	**19**	**11.38 (2.63)**	**6.93(1.84)** ^**b**^	**6.79 (2.14)** ^**b**^	**7.68(2.11)** ^**b**^	**2 M2, 17 M3**

1. The text should be corrected to indicate that there were 14, not 13, girls with FXS-ASD at time two (column 2)
2. The standard deviation for chronological age for the boys with FXS was inadvertently left out [column 3 (Chronological Age)].
3. The frequency of module distribution should be corrected for boys with ASD at time one [last column (“ADOS Module Distribution)].

Changes to text (appearing on page 4, beginning of Results section):

The text below also incorrectly reported the percentage of girls meeting ASD criteria and overall proportion of individuals with FXS meeting criteria for ASD at time one. This text should read:

- “Overall, 41.5% of children with FXS met criteria for ASD at time one (54.8% of boys, 29.4% of girls); at time two, 60% of the sample met criteria (80.6% of boys, 41.2% of girls), a statistically significant change (*p* = .008), driven by the change in classification in boys with FXS (*p* = .021).”
